# Post‐mortem interval determinations using insects collected from illegally hunted and dehorned rhinoceros in the Republic of South Africa from 2014 to 2021

**DOI:** 10.1111/mve.12760

**Published:** 2024-10-09

**Authors:** Melanie Pienaar, Ian R. Dadour

**Affiliations:** ^1^ Forensic Science Laboratory Victim Identification Center Pretoria South Africa; ^2^ Source Certain International Wangara Western Australia Australia; ^3^ Discipline of Medical, Molecular & Forensic Sciences Murdoch University Murdoch Western Australia Australia

**Keywords:** forensic entomology, minimum PMI, Republic of South‐Africa, rhinoceros poaching cases

## Abstract

Wildlife forensic science is a growing research field globally with application in criminal cases of illegal hunting requiring an estimate of time of death based on insect fauna. The techniques and procedures of forensic entomology acquired over the last 40 years, used in legal cases relating to human remains, can be adapted to decomposing wildlife. Research on carrion utilising the rate of development of insect immatures provides a biological clock from which a minimum post‐mortem interval (minPMI) can be derived. The following study concerns 19 rhinoceros that were illegally killed and dehorned in the Republic of South Africa between 2014 and 2021. The paper details 74 samples of insect evidence collected from these rhinoceros remains from which an accurate estimate of their PMI was calculated. The specimens comprised 18 species from 12 families belonging to three insect orders. Many Dipteran and Coleopteran species were found on and around each carcass. The species of fly larvae (family Calliphoridae) used in each case to estimate the PMI are as follows: *Chrysomya marginalis* (Wiedemann) (13 cases), *Chrysomya chloropyga* (Wiedemann) (2 cases), *Chrysomya albiceps* (Wiedemann) (1 case) and *Chrysomya megacephala* (Fabricius) (1 case). Two species of Coleoptera from the family Dermestidae and Silphidae involved *Dermestes maculatus* (DeGeer) and *Thanatophilus micans* (Fabricius), respectively, also were involved in one PMI estimation each. The paper highlights opportunities for improving our global understanding of gaps in procedures and training related to wildlife criminal cases.

## Graphical abstract

- A total of 6684 rhinoceros were poached in South Africa during 2014 to 2021.
- Insect evidence collected from 19 poached rhinoceros was analysed by the Forensic Entomology laboratory of the South African Police Service.
- Six species of dipterans and coleopterans were utilised to determine an accurate estimate of the minimum post‐mortem interval.

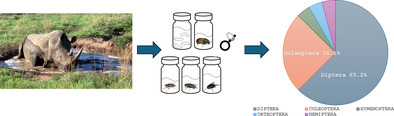

## Abstrak

Die gebruik van insekte om die nadoodse interval van onwettig gejag en onthoringde renosters wat in die Republiek van Suid‐Afrika van 2014 tot 2021 versamel is, te bepaal

Wild forensiese wetenskap is ‘n groeiende navorsingsveld wêreldwyd met toepassing in kriminele gevalle van onwettige jag wat ‘n skatting van tyd van dood vereis gebaseer op insekfauna. Die tegnieke en prosedures van forensiese entomologie wat die afgelope 40 jaar verkry is, wat in regsake met betrekking tot menslike oorskot gebruik is, kan aangepas word by die ontbinding van wild. Navorsing oor aas wat die tempo van ontwikkeling van insek‐onvolwassenes gebruik, bied ‘n biologiese horlosie waaruit ‘n minimum nadoodse interval (minPMI) afgelei kan word. Die volgende studie handel oor 19 renosters wat tussen 2014 en 2021 onwettig in die Republiek van Suid‐Afrika doodgemaak en onthoring is. Die koerant bevat 74 monsters van insekbewyse wat uit hierdie renosterreste versamel is, waaruit ‘n akkurate skatting van hul PMI bereken is. Die monsters bestaan uit 18 spesies uit 12 families wat tot 3 insekordes behoort. Baie Diptera‐en Coleoptera‐spesies is op en om elke karkas aangetref. Die spesies vlieglarwes van die Calliphoridae familie wat in elke geval gebruik word om die PMI te skat, is soos volg: *Chrysomya marginalis* (Wiedemann) (13 gevalle), *Chrysomya chloropyga* (Wiedemann) (2 gevalle), *Chrysomya albiceps* (Wiedemann) (1 geval) en *Chrysomya megacephala* (Fabricius) (1 geval). Twee spesies Coleoptera van die familie Dermestidae en Silphidae onderskeidelik was *Dermestes maculatus* (DeGeer) en *Thanatophilus micans* (Fabricius), wat ook betrokke by was in 1 PMI skatting elk. Die referaat beklemtoon geleenthede vir die verbetering van ons globale begrip van leemtes in prosedures en opleiding wat verband hou met wildmisdadige sake gebaseer op die evaluering van plaaslike wildlewe‐gevallestudies.

## INTRODUCTION

Since the 1990s, increasing attention has been paid to the roles forensic science can play in addressing legal cases related to trade or criminal use involving wildlife (Anderson, 1999; Merck, 2013). Early applications of forensic science have been steadily widening, in both wildlife and veterinary fields. Despite this, the use of entomological perspectives, quality‐controlled standard procedures and training to support wildlife and veterinary forensic cases has not been optimised (Gouda et al., 2020). Crimes such as poaching for bushmeat consumption and illegal trade for ornamental commercial items, pets or traditional medicine ingredients threatening wildlife species worldwide often cross jurisdictional borders. Despite the formation in 2010 of the International Consortium on Combating Wildlife Crime (ICCWC), more than 70% of the cases of wildlife crimes remain unsolved due to the inappropriate identification of crime evidence (Baptista et al., 2022).

Publication and subsequent peer review of geographically specific case studies about criminal use and trade of wildlife are helping practitioners to identify and address the knowledge, practice and training gaps presently constraining wider adoption of wildlife forensic science practices. In all but a few cases (Munro & Munro, 2008), the approaches used are entirely based on those developed for cases involving the application of forensic entomology to human criminal cases. In support of such practices, there are many excellent published texts on forensic entomology and the methodologies used for human applications, regardless of the geographical jurisdiction (Amendt et al., 2007). However, despite these practices, many deficiencies still occur mainly due to entomological evidence being collected by crime scene technicians rather than by an experienced forensic entomologist (Bambaradeniya, Magni, & Dadour, 2023).

In Southern Africa, it is very unlikely to find a death scene without insect activity present. This is due to the temperate climate of the region and the attraction of numerous insect species to decaying organic material such as human or other remains (Benecke, 1998; Catts & Haskell, 1991). Insect species visit carrion in a predictable pattern depending on the group's feeding requirements, collectively referred to as sarcosaprophagous fauna (Arnaldos et al., 2005; Payne, 1965). A phase of undisturbed exposure follows death prior to insects detecting remains and is referred to as the pre‐colonisation period (Reibe & Madea, 2010; Tomberlin et al., 2011; Villet, 2011). Following this period, blowfly species (Diptera) and beetles (Coleoptera) are generally the first groups of insects to colonise remains after death (Magni et al., 2015). The females usually lay eggs, and the subsequent life cycle acts as a biological clock (Greenberg & Kunich, 2002). When the development period concerning each stage of the insect life cycle is known, one can estimate the minimum post‐mortem interval (minPMI) (Aggarwal et al., 2003; Villet & Amendt, 2011). Consequently, apart from taxonomy, the largest body of research has focussed on the development data of many forensically relevant blowfly species (Bambaradeniya, Magni, Dadour, & Weldon, 2023). These data are used to estimate the PMI via three types of calculation: accumulated degree day/hour (ADD/ADH), tables of growth and isomegalen/ isomorphen diagrams (Bambaradeniya, Magni, & Dadour, 2023; Harvey et al., 2016; Richards, Williams, & Villet, 2009). The ADD/ADH method is the most common technique compensating for the development data generated in controlled environments in the laboratory and then applied to fluctuating temperatures experienced at crime scenes (Zalom et al., 1983).

Rhinoceros poaching is a reality in The Republic of South Africa (RSA), with large numbers (Table 1) of animals killed for their horns, which are removed and sold for medicinal purposes or as a sign of wealth in certain Asian markets. The RSA contributes approximately 90% of all rhinoceros horns entering the illegal trade markets (Rhino poaching and illegal trade decline but remain critical threat–new report, 2023, November, 06). In 2011, forensic entomology was established in the Division of Forensic Services of the South African Police Service (SAPS) to report on the minPMI of human remains. Three years later, in 2014, it was adopted to report on rhinoceros poaching cases, whereby insect evidence was collected and analysed to estimate the minPMI of these animal remains.

**TABLE 1:** A comparative summary of the number of rhinoceros poaching cases (2014–2021) reported from all South African National Parks, including the Kruger National Park to the number of these rhinoceros remains (*) from which insect evidence was collected for entomological analysis across all nine provinces.

National parks & provinces	2014	2015	2016	2017	2018	2019	2020	2021	Totals
SANParks KNP	827	826	662	504	422	327	245	209	4022
SANParks other	1	0	0	0	2	1	2	0	6
Gauteng	5	2	6	4	2	5	2	2	28
Limpopo	110	91	90	79	40	45	18	38	511
Mpumalanga	83	67	32 (*1)	49	51	34	13	39	368 (*1)
North West	65	46	56	96	65	32 (*2)	19	32	411 (*2)
Eastern Cape	15 (*1)	14	19	12	19	2 (*2)	0	0	81 (*3)
Freestate	4	10	17 (*2)	38	16 (*3)	11	1 (*1)	24 (*6)	121 (*12)
Kwazulu Natal	99 (*1)	116	162	222	142	133	93	102	1069 (*1)
Western Cape	1	1	0	0	0	0	0	4	6
Northern Cape	5	2	12	24	12	4	1	1	61
TOTALS	1215 (*2)	1175	1056 (*3)	1028	771 (*3)	594 (*4)	394 (*1)	451 (*6)	6684 (*19)

This study reports on the insect evidence obtained from the remains of 19 rhinoceros that were poached across five provinces of the RSA between 2014 and 2021. The first objective focussed on the identification of the species involved and the second objective was to tabulate the best estimate for the minPMI in each case.

## MATERIALS AND METHODS

**FIGURE 1:**
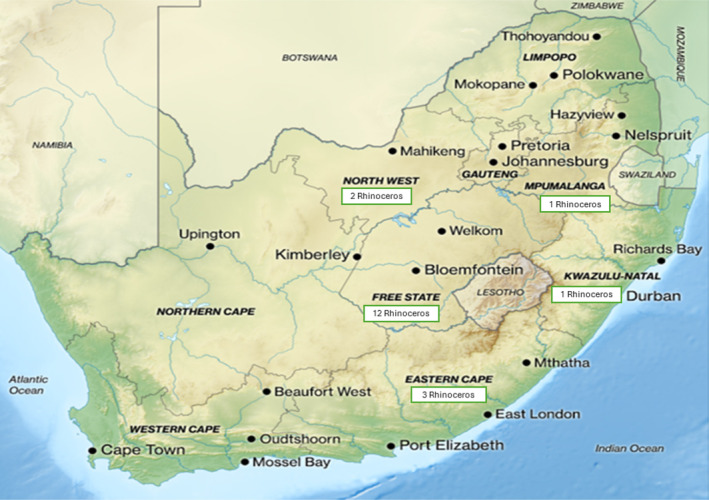
Map of the RSA indicating the number of rhinoceros remains per province from which insect evidence was collected in this study. Adapted from South Africa relief location map (Gateway, n.d.).

During this study (2014–2021), a total of 6684 illegally hunted and poached animals were reported from across the nine provinces of the RSA (Table 1). The judicial system in the RSA mandates that strict insect collection protocols be followed to ensure a proper chain of custody and to secure samples of sufficient quality for identification and the estimation of an accurate minPMI. Over the last decade, 213 crime scene technicians from across the RSA have been issued with an entomology collection kit and received basic training in the procedures of collecting insect evidence from crime scenes involving both human and rhinoceros remains (Amendt et al., 2011; Bambaradeniya, Magni, Dadour, & Weldon, 2023). Over the last 7 years, the forensic entomology laboratory has analysed insect evidence collected from 19 poached rhinoceros remains. This study involved the remains of 18 adults and one calf. From the 19 remains, a total of 74 insect samples were collected, which included 119 individual insects, both dead and alive, across all stages of development (Table 2). The rhinoceros remains were from five provinces in the RSA: North West, Free State, Eastern Cape, Mpumalanga and Kwazulu Natal (Figure 1).

**TABLE 2:** Summary of the total number of insect specimens for each species collected from rhinoceros carcasses from five provinces of the RSA during all four season.

Identified specimens	Summer		Autumn			Winter	Spring		Total
	FS	EC	FS	NW	MP	FS	FS	KZN	
**Order: Diptera**									**89**
*Chrysomya marginalis*	5	11	6	5	1	1	2	1	32
*Ch. albiceps*	3	5	4	6	1	1	2		22
*Ch. chloropyga*			2			3	3		8
*Ch. putoria* Wiedemann				2					2
*Ch. megacephala*								2	2
*Lucilia cuprina* Wiedemann				1			2	1	4
*Hydrotaea capensis* Wiedemann				1		1	1		3
*Musca spp*		2	3			1	1		7
*Sepsidae spp*			1		1				2
*Piophila casei* Linnaeus		5		1					6
*Asilidae spp*					1				1
**Order: Coleoptera**									**28**
*Histeridae spp*	3	1			1			1	6
*Dermestes.maculatus*	5	4							9
*Thanatophylus.micans*	4	2							6
*Necrobia rufipes* De Geer	2	2							4
*Trogidae spp*					1				1
*Scarab spp*		1						1	2
**Order: Hemiptera**									**2**
*Scuteleridae spp*	2								2
**Total**									**119**

Abbreviations: FS, Free State; EC, Eastern Cape; KZN, Kwazulu Natal; MP, Mpumalanga; NW, North West.

### Scene collection

Once a dead rhinoceros had been discovered, the following procedure ensued. It was reported to the provincial Environmental Management Inspectorate (EMI) who then established if any criminal activity had occurred. The SAPS then became involved once the remains were determined as poached. A death scene involves numerous authorised personnel from many disciplines who are tasked with the retrieval and preservation of evidence. This includes but is not limited to physical items of potential forensic significance, DNA, fingerprints and insects (Catts & Haskell, 1991). A necropsy was completed at the scene by a veterinarian to determine the manner and cause of death. Other related evidence (projectiles or darts, calibre of the firearm used, trajectory and tool marks of hacking blades, saws or axes) also were determined and collected.

The entomological investigation at the death scene was subdivided into four parts. The initial part involved observation and notation of the environmental conditions, the ecological characteristics of the scene, the condition of the remains (e.g. stage of decay, predation, dismemberment and trauma) and the insect activity on and around the remains. Meteorological data and temperatures directly affecting insect development (e.g. larval mass temperature) also were recorded at each site (Byrd & Tomberlin, 2019; Catts & Haskell, 1991). The second part involved photographic documentation at each crime scene. Photographs of the location, carcass and observable insect activity were recorded. The third part involved the collection of insect specimens, which were divided into preserved and live samples. Samples of all development stages of fly and beetle specimens were collected and preserved. These samples were used to determine the physiological age of the insects as well as the species composition of insects at the time of collection (Villet & Amendt, 2011). Adult flies were collected with a sweep net, sprayed with 90% alcohol to immobilise them and preserved in 70–80% alcohol. Fly larvae and full puparia, adult beetles, beetle larvae and pupae were collected and immersed in hot water (70–80°C) for 1 min prior to preservation. The casings of live fly puparia were pricked twice with a surgical needle after immersion in hot water to allow the alcohol to penetrate and preserve the internally developing fly. Collected eggs and empty puparia were placed directly in 70–80% alcohol for preservation. A live sample of all immature fly specimens (eggs, larvae and full puparia) was then collected. Eggs and larvae were kept in a ventilated container with a moist paper towel to prevent desiccation. A food source (chicken liver) was added to the container should the samples not reach the forensic entomology laboratory within 24 h after collection. Living full fly puparia were placed in a ventilated container with a dry paper towel. These samples are kept alive for rearing and identifying emerging adults. The fourth part included compiling a report containing all the information relevant to the stage of decay, the species composition and succession, life history stages of the immatures and potential limitations that may impinge on calculating an accurate minPMI.

### Laboratory protocols

The insect evidence was analysed within 48 h following collection. Insect specimens were delivered to the forensic entomology laboratory in Pretoria, Gauteng Province (RSA). Chain of custody of all samples from the scene to the laboratory is assured by using a barcoded system. Overall, a total number of 60 preserved and 14 live samples were collected from the 19 rhinoceros.

Live samples from the scene were transferred onto a fresh chicken liver medium and then placed onto bedding material (e.g. vermiculate or dry sand) inside a ventilated container to rear to adult. All samples were kept under natural lighting conditions and fluctuating temperatures. The RSA transitions between four seasons: summer from December to February, autumn from March to May, winter from June to August and spring from September until November. The climatic zones of RSA vary and include deserts, tropical forests and montane grassland. Significant temperature and humidity differences exist between these zones (Richards & Villet, 2009). The temperatures in the laboratory simulated the average seasonal temperatures across the five provinces: a minimum temperature of 19°C and a maximum of 30°C in summer and a minimum of 7°C (±2°C) and a maximum of 21°C (±2°C) in winter. (Africa, 2023). Across the RSA, the daily average hours of sunshine vary between 8 to 9.5 h. The lighting conditions at which samples were reared in the forensic entomology laboratory were 9‐h light and 15‐h dark. Enclosed adult flies following full pigmentation and wing formation were preserved in 70–80% alcohol and identified.

### Identification and age determination

In order to estimate the minPMI, immature and adult insect specimens from all received samples were identified using a Leica EZ4 HD digital stereo microscope and a Lasec UB103i biological microscope. Appropriate identification keys of species were consulted (Brink, 2009; Irish et al., 2014; Lutz et al., 2018; Midgley et al., 2010; Williams & Villet, 2014; Zumpt, 1965). The approximate age of the larvae in each sample was determined by measuring their length and utilising other morphological features (e.g. the posterior spiracle or cuticular banding patterns) (Adams & Hall, 2003; Catts & Haskell, 1991). The minPMI was then estimated by using the development stage of the immature insects at the time of collection, and then cross‐referencing with the temperature and development data available for these species. (Lunt, 2002; Magni et al., 2015; Midgley & Villet, 2009; Prins, 1982; Richards, Williams, & Villet, 2009; Richardson & Goff, 2001; Ridgeway et al., 2014; Zumpt, 1965).

### PMI estimation

The thermal summation model was applied in this study to estimate the insect development rate. The life cycle of an insect species is measured in physiological time and refers to the amount of heat required by an insect to complete its various life stages. The identified species used for minPMI estimations relied on published development data as follows: *Chrysomya marginalis* (Wiedemann), (Lunt, 2002; Richards, Price, & Villet, 2009), *Chrysomya chloropyga* (Wiedemann) (Lunt, 2002; Richards, Crous, & Villet, 2009; Richards, Price, & Villet, 2009), *Chrysomya albiceps* (Wiedemann) (Lunt, 2002; Richards et al., 2008; Richards, Price, & Villet, 2009), *Chrysomya megacephala* (Fabricius) (Lunt, 2002; Richards, Price, & Villet, 2009; Richards & Villet, 2009), *Dermestes maculatus* (DeGeer) (Magni et al., 2015; Richardson & Goff, 2001) and *Thanatophylus micans* (Fabricius) (Midgley & Villet, 2009; Ridgeway et al., 2014). A degree day occurs when the average temperature over 24 h is 1° above the lower development threshold (LDT) and below the maximum development threshold temperature required for insect development (Amendt et al., 2007; Bambaradeniya, Magni, & Dadour, 2023; Bambaradeniya, Magni, Dadour, & Weldon, 2023; Harvey et al., 2016; Lunt, 2002). Hourly temperatures within the minimum and maximum thresholds may also be used for summing the actual amount of heat accumulated each day or over a specific time period. The formula used is ADD/ADH = Time × (Average Temperature across the time period – LDT) for the species. The time elapsed between oviposition and sample collection was calculated considering the temperatures recorded at the crime scene (Harvey et al., 2016).

Hourly weather conditions (e.g. ambient temperature, rainfall, relative humidity and cloud coverage) for each crime scene involving a rhinoceros were obtained from the nearest South African Weather Services station and compared to those recorded at the scene. Contextual information pertaining to each criminal case is privy to judicial permission and is not presented in this study. Consequently, the following procedure to determine each minPMI is as follows and is detailed in Table 3. The species of fly was identified, the stage of development noted, the length of the largest larvae for each case was measured, and this combined with the optimal development temperature the minPM was determined. The optimal development temperature was used by the forensic entomologist because although not measured, the larval masses were evident from the photographs in each case.

**TABLE 3:** Summary of the reported data including the minimum post mortem interval (minPMI) estimation from the insect evidence collected from 19 rhinoceros remains in the RSA across five provinces and four seasons.

Season	Province	Species	Stage of development	Sample condition	Length (mm)	Stage of decay	Ambient temperature at collection (°C)	Larval mass temperature (°C)	Predation	Exposure type	Average ambient temperature during PMI period	minPMI estimate (days)
	FS	*Chrysomya. marginalis*	Third instar	P	14	Active	28.2	33.7	Y	SS	30.6	2
	FS	*Ch. marginalis*	Pupae	P	N/A	Advanced	19.1	27.8 (soil)	Y	S	24.2	12
	FS	*Ch. marginalis*	Third instar	P	14	Advanced	34.4	38	Y	SS	24.4	5
	FS	*Ch. albiceps*	Third instar	P	11	Advanced	33.3	38	Y	SS	27.6	7
SUMMER	FS	*Thanatophylus micans*	Larva	P	9	Advanced‐Dry	34.4	X	Y	SS	27	20
	FS	*Ch. marginalis*	Third instar	P	18.7	Advanced	30.5	40.6	N	SS	34	3
	EC	*Dermestes maculatus*	Pupae	P	N/A	Dry remains	29	29 (soil)	N	S	24.3	90
	EC	*Ch. marginalis*	Third instar	P	9.6	Active	27	28	Y	S	27.5	2
	EC	*Ch. marginalis*	Third instar	P	10	Bloated‐Active	36	41	N	S	28.8	3
	FS	*Ch. marginalis*	Third instar	P	20	Bloated‐Active	37	41	N	S	27.1	2
	FS	*Ch. marginalis*	Third instar	P	18	Active	34	41	Y	SS	24	4
AUTUMN	FS	*Ch. chloropyga*	Pupae	L	N/A	Advanced	14.2	25.6	N	SS	9	11
	NW	*Ch. marginalis*	Third instar	P	13.5	Bloated‐Active	19.3	40.1	N	S	21.2	5
	NW	*Ch. marginalis*	Third instar	P	13.1	Bloated‐Active	19.9	39.6	N	S	19.2	4
	MP	*Ch. marginalis*	Third instar	L	12.4	Active‐Advanced	33	38	N	SS	31.6	2
WINTER	FS	*Ch. chloropyga*	Post feeding	P	7.7	Active	27.4	34.2	Y	S	15.8	8
	FS	*Ch. marginalis*	Third instar	P	15	Active	30	39	Y	S	30.2	4
SPRING	FS	*Ch. marginalis*	Third instar	P	15.3	Active	30	39	Y	S	30.2	4
	KZN	*Ch. megacephala*	Third instar	P	15	Bloated	22	39	N	S	20.8	5

Abbreviations: EC, Eastern cape; FS, Free State; KZN, Kwazulu Natal; L, Live; MP, Mpumalanga; N, No; NW, North West; P, Preserved; S, Full sun; SS, Semi‐shade; Y, Yes.

## RESULTS

### Specimen summary

Dipteran species represented 61% of the total number of collected specimens. A total of 18 insect species across three insect orders (Diptera, Coleoptera and Hemiptera) were identified from the 74 insect samples received (Table 2). The Hemiptera species collected were of no evidential value regarding the time of death aspects. In most cases, individuals from this order are found to be adventive or incidental species with only some having the potential to provide evidential value (Villet, 2011). This study only focussed on the six species from Diptera and Coleoptera, which were of forensic significance and used for the 19 minPMI estimations in this study (Table 3). Four Calliphoridae species used for the minPMI estimations were identified as *Ch. marginalis*, *Ch. chloropyga*, *Ch. albiceps* and *Ch. megacephala*. *Ch. marginalis* was the most prevalent calliphorid present during the warmer climatic conditions across all five provinces. Numerous *Ch. albiceps* specimens also were examined but only used in 1 min PMI estimation. *Ch. chloropyga* was the dominant species during the cooler seasons and was used in 2 min PMI estimations.

Coleopteran species were present and constituted 33% of the collected carrion community from the rhinoceros remains. The two species, Dermestidae: *D. maculatus* and Silphidae: *T. micans*, were used in two minPMI estimates. These specimens were preserved samples of coleopteran pupae and larvae, respectively (Table 3). *D. maculatus* and *T. micans* specimen*s* were used in 1 min PMI estimation each and resulted in a reliable PMI estimation in both investigations when cross‐referenced with other non‐insect evidence.

### Meteorological data

Due to the rural localities of all the scenes in this study, the nearest weather station was not always in close proximity. In the Free State, the nearest stations ranged from 10 to 55 km for nine crime scenes, while the remaining three cases were approximately 70, 150 and 180 km from the weather stations. In the Eastern Cape, the distance between the weather stations and the three crime scenes was 42, 77 and 78 km. The two rhinoceros carcasses from North West province were from the same location, 18 km from the weather station. The nearest station to the single Mpumalanga and Kwazulu Natal crime scenes was 10 km away. However, since most temperatures recovered from every scene were limited to a single data point (63% of cases), inevitably the weather data was used in ADD calculations.

The seasonal climate of the various provinces recorded by the weather stations indicated that the highest and lowest ambient temperatures for Free State were 40°C during mid‐summer and −7.2°C during early winter, respectively. The maximum ambient temperature of the Eastern Cape summer cases was recorded at 36.5°C, and the lowest was 4.2°C. The maximum ambient temperatures for the single cases from Kwazulu Natal during spring, Mpumalanga and North West during autumn were documented as 35.9, 39.3 and 29.3°C, while the minimum temperatures for these provinces during the estimated PMI periods for the respective rhinoceros remains were recorded as 11.6, 18°C and 10.1°C.

The temperature of larval masses was measured and recorded at the scene during the collection process. The highest larval mass temperature frequently recorded for *Ch. marginalis* ranged between 37.3 and 41°C compared with the recorded scene ambient temperatures that ranged between 19.3 and 37°C. The range of larval mass temperatures recorded for *Ch. chloropyga* was 25.6 and 34.2°C when the ambient temperature was 14.2 and 27.4°C, respectively. Larval masses formed by *Ch. megacephala* produced temperatures of 39°C when the ambient temperature was 22°C while the larval masses of *Ch. albiceps* were 38°C when the ambient temperature was 33.3°C.

The degree of exposure to the natural elements (sun, humidity and rain) following the discovery of the carcasses was considered when calculating the rate of development of the various specimens used in the minPMI estimations. Eleven carcasses were exposed to full sun. This included five remains in the Free State, all three carcasses in the Eastern Cape, both carcasses in the North West, and the single carcass in Kwazulu Natal. Eight carcasses were discovered in semi‐shade conditions, of which seven were exposed to semi‐shaded conditions in the Free State as well as the single case from Mpumalanga. No rainfall, humidity or wind speed was recorded in any of the cases included in this study.

### Stage of decay and scavenger activity

From the scene reports and the photographic images received, the stage of decay of the 19 rhinoceros remains in the respective provinces was as follows: in the Free State, one carcass was in transition between bloated to active, five were in active decay, five were in advanced decay while one was in transition between advanced to dry decay. The three carcasses in the Eastern Cape were in transition between bloated to active, active, and dry decay, respectively, while the two carcasses in North West were both in transition between bloated to active decay. The single carcass collected from Kwazulu Natal was bloated, while the carcass in Mpumalanga was in transition from active to advanced decay.

Although not observed, lions, hyenas, jackals, and vultures were the main scavengers causing soft tissue damage to the carcasses. In the Free State, nine of the 12 carcasses, and in the Eastern Cape, one of the three carcasses sustained damage by scavengers. Carcasses from the other provinces included in this study showed no evidence of large mammal scavenging.

### PMI estimations

A total of 13 PMI estimations were calculated using the development stage of *Ch. marginalis* specimens. These specimens from the Free State included seven preserved third instar larvae and a preserved pupa. Two preserved specimens of third‐instar larvae from the North West and the Eastern Cape were used, respectively, while one preserved third‐instar larvae of *Ch. marginalis* from the Mpumalanga carcass was used. *Chrysomya chloropyga* was used in two PMI estimations. Both were from the Free State and consisted of one specimen of live pupae and one specimen of preserved post‐feeding larvae, respectively. The PMI estimations using *Ch.albiceps* and *Ch. megacephala* included one preserved specimen of third‐instar larvae each. The *Ch. albiceps* specimen was collected from rhinoceros remains in the Free State and the *Ch. megacephala* sample was from Kwazulu Natal. The two coleopteran species used for timeline estimations included one preserved specimen of *D. maculatus* pupae and one preserved *T. micans* larvae collected from carcasses located in the Eastern Cape and the Free State, respectively (Table 3). The larval mass temperatures did not exceed the upper development threshold for all 19 cases (Table 3) and hence, were used in all ADD calculations.

## DISCUSSION

The results of the minPMI in this paper relied on the published information from studies regarding colonisation, succession, development rates and identification of fly species (Braack, 1981; Brink, 2009; Ellison, 1990; Irish et al., 2014; Lunt, 2002; Lutz et al., 2018; Prins, 1982; Richards et al., 2008; Richards, Crous, & Villet, 2009; Richards & Villet, 2009; Villet, 2011; Williams & Villet, 2014; Zumpt, 1965).

In a forensic context, the physiological age of calliphorid flies is ideal for calculating minPMI estimations. The four blowfly species collected from 17 rhinoceros cases included *Ch. marginalis*, *Ch. albiceps*, *Ch. chloropyga* and *Ch. megacephala*. *Chrysomya marginalis* was the dominant species during the warmer seasons, while *Ch. chloropyga* was the dominant species during the cooler months. This seasonal preference is consistent with the thermo‐physiological tolerances of the two species (Richards, Price, & Villet, 2009; Richards, Williams, & Villet, 2009). However, only limited data for the development threshold temperatures of *Ch. marginalis* currently exist. The average upper lethal development temperature and average LDT for *Ch. marginalis* are indicated as 50.1 and 14.0°C, respectively, while those of *Ch. chloropyga* are 49.2 and 10.9°C (Richards, Price, & Villet, 2009).

Conclusions regarding *Ch. marginalis* were drawn from information on the species scattered throughout the literature. Most significantly were the biological notes by Prins (1982) and Meskin (1980), which provided valuable information on incubation time in relation to succession as well as seasonal development times of the larvae and pupae. The box plots of the species by (Richards & Villet, 2009) furthermore supplied data on some temperature thresholds. The baseline development data for this species also were detailed in the study by Lunt (2002). This included the mean development times for all *Ch. marginalis* life history stages (larval instars and pupae) at 25°C and was subsequently used to calculate the ADD.

Within the various life history stages, the physiological age of any specific specimen is estimated by its developmental maturity (Villet, 2011). Larval length is not the most effective indication of the physiological age of a specimen (Dadour et al., 2001). The diagnostic descriptions for the immature stages of *Ch. marginalis* as detailed by Brink (2009) clearly highlight the progressive morphological changes (e.g. the cephalopharygeal skeleton, the posterior spiracles and the spiracular plate) of the species as it matures within each life stage towards a developmental milestone. This information in conjunction with the known development period for each life stage and taking into account the temperatures the specimen had been exposed to provide the information to determine the physiological age of the specimen at the time of collection at the scene. Once this age is known one can successfully calculate the minPMI using the ADD/ADH method as presented in Table 3.

Immature *Ch. albiceps* were present on 15 of the 19 rhinoceros remains but only used in one minPMI. This was because it is a secondary coloniser, generally with a 1–2 days delay in infestation compared with the primary colonising species. This confirms the observations and findings of earlier studies. (Braack, 1987; Prins, 1982; Smith, 1986; Zumpt, 1965).

The RSA has the highest rhinoceros poaching rate in Africa (Seasons of the Year, 2023) and a homicide rate of 33.5/100000 individuals per annum (Damons & Geffen, 2023, 2 June). This makes it impossible for the entomologist to attend every death scene or despoilment of wildlife. The increasing demand for entomological input (Kotzé et al., 2021) from the judiciary in a death investigation has resulted in the ongoing training of 213 crime scene technicians in all nine provinces of the RSA. Like many law enforcement agencies, crime scene technicians are generally transitory, and the skill base wanes, so this consistent training is critical so that entomological evidence will become more reliable, allowing the forensic entomologist to make the best estimate of a minPMI (Morris et al., 2015; Richards & Villet, 2008). Since establishing the forensic entomology laboratory in 2011, the SAPS has analysed and reported on insect evidence collected from 220 human and 44 rhinoceros death scenes. This study on rhinoceros remains only included those collections with complete data and preserved samples to produce reliable PMI estimations (Bambaradeniya, Magni, Dadour, & Weldon, 2023). Unfortunately, the insect evidence collected from many rhinoceros carcasses has been excluded due to poor protocols concerning collecting and preserving insect material and the neglect to record the temperatures at the scene.

The forensic entomologist typically uses both live and preserved samples of immature specimens. All insect evidence collected from a crime scene in RSA is sent to the laboratory in Pretoria, Gauteng. However, this is often delayed for extended periods due to limited transport from remote stations and the material becomes degraded. Unfortunately, poorly preserved samples affect important taxonomic features (Kavitha et al., 2013) and age determination. When possible, reliable identification keys for Diptera and Coleoptera larvae and adults are used to identify the collected specimens to species level (Brink, 2009; Irish et al., 2014; Lutz et al., 2018; Prins, 1982; Williams & Villet, 2006; Williams & Villet, 2014; Zumpt, 1965). Currently, the use of DNA for identifying species is not readily available in the RSA (Harvey et al., 2003; Harvey et al., 2016; Villet, 2011).

Blowflies are ectotherms whose development, behaviour and physiology result from the temperatures they experience (Harvey et al., 2016; Richards, Williams, & Villet, 2009). In many of the rhinoceros cases, there was an absence of measured ambient scene temperatures, so the only data used were that received from the nearest weather station, and this is not always conducive to an accurate minPMI estimate (Catts, 1992; Megyesi et al., 2005).

Furthermore, larval aggregations produce temperatures that can exceed ambient temperatures and produce optimal heat levels for fly development (Catts, 1992; Kotzé et al., 2016; Lunt, 2002). When technicians or proxies make collections, they generally overlook measuring the temperatures from larval masses. In this study, larval masses were only observed from the scene photographs. In the absence of this temperature and based only on these photographs of larval masses present on the rhinoceros carcasses, the optimal development temperatures at the relevant development stages for each species were used (Lunt, 2002; Richards et al., 2008; Richards & Villet, 2009).

Since 2000, in the RSA, the criminal justice system has recognised that insect evidence is forensically valuable (Williams & Villet, 2006), even though it is not always collected at the gold standard level (Marks et al., 2015). Every case involving forensic entomology is reported on in terms of Section 212 of the RSA Criminal Procedure Act, 1977 (Act 51 of 1977),  (Section 212 of the RSA Criminal Procedure Act, 1977). Reports written by the forensic entomologist are not scientifically peer‐reviewed papers but are compiled to simplify the findings for the court (Kotzé et al., 2021). The court accepted the forensic entomology reports for all 19 cases without challenge, so it may be concluded that the life history data for all species derived from the various published studies and then converted to a minPMI were considered reliable. Determinations must be transparent and impartial, including all caveats relating to the minPMI estimation. This forensic report if accepted by the judiciary in the RSA as testimony does not necessitate the presence of the forensic entomologist to present the evidence in court. Finally, forensic entomology expert witness statements were presented in court by the forensic entomologist for two of the 19 cases involved in this study. The summation of all evidence including the forensic entomology report presented to the judge resulted in one perpetrator receiving a collective sentence of 24 years of imprisonment.

The RSA has been active in rhinoceros conservation over the last 30 years, resulting in a rebound in rhinoceros populations (Seasons of the Year, 2023). However, the black rhino remains in the top 10 of the International Union for Conservation of Nature's red list of critically endangered species (Rhino poaching and illegal trade decline but remain critical threat‐new report, 2023, 6 November). This fact has not gone unnoticed in the RSA with every effort now made to stop rhinoceros poaching, including the development of ranger teams, canine units and satellite tracking (Rhino poaching and illegal trade decline but remain critical threat‐new report, 2023; Seasons of the Year, 2023).

The high incidence of illegal killing of rhinoceros in the RSA has provided a sad but unique opportunity to examine the effectiveness of forensic entomology practices in supporting those who have a responsibility for estimating time of death of the wildlife and constructing a case against suspected perpetrators. There are few other locations in the world that provide forensic entomologists ongoing access to such rich, contextual information on insect species, environmental conditions and scene collection processes, complemented by government and institutional support for growing the application and effectiveness of wildlife forensic science.

## AUTHOR CONTRIBUTIONS

**Melanie Pienaar:** Conceptualization; methodology; data curation; investigation; project administration; resources; writing – original draft; writing – review and editing. **Ian R. Dadour:** Methodology; validation; supervision; formal analysis; writing – original draft; writing – review and editing.

## FUNDING INFORMATION

This project received no funding, but was supported by the South African Police Services, Forensic Science Laboratory.

## CONFLICT OF INTEREST STATEMENT

The authors report there are no competing interests to declare.

## ETHICS STATEMENT

This study did not involve any human subjects and permission to carry out and compile this research was granted by the Head of Research, South African Police Service The research was conducted in accordance with South African Police Service, National Instruction 4 of 2022 guidelines to share data on rhinoceros poaching.

## Supporting information


**DATA S1:** Structured Reflexivity Statement.

## Data Availability

Research data are not shared.
