# Supplementary material for: Post‐mortem interval determinations using insects collected from illegally hunted and dehorned rhinoceros in the Republic of South Africa from 2014 to 2021
Source: Med Vet Entomol. 2024 Oct 9;39(1):58–68. doi: 10.1111/mve.12760 (PMC11793131; doi:10.1111/mve.12760)

Structured Reflexivity Statement

Th research was caried out in South Africa by Capitan M Pienaar. There was no funding for the work other than this was and is part of her job. The input by Dr Dadour was in an advisory role and no funding was granted. This collaborative manuscript has been a 3 year process, an is the result of a FE workshop in Pretoria, South Africa in 2013.


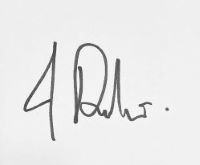

Supplement: Supplementary file 1 — DATA S1: Structured Reflexivity Statement. [file MVE-39-58-s001.docx]
